# Female sex hormones and symptoms of obstructive sleep apnea in European women of a population-based cohort

**DOI:** 10.1371/journal.pone.0269569

**Published:** 2022-06-22

**Authors:** Erla S. Sigurðardóttir, Thorarinn Gislason, Bryndis Benediktsdottir, Steinar Hustad, Payam Dadvand, Pascal Demoly, Karl A. Franklin, Joachim Heinrich, Mathias Holm, Diana A. van der Plaat, Rain Jõgi, Benedicte Leynaert, Eva Lindberg, Jesus Martinez-Moratalla, Leire Sainz De Aja, Giancarlo Pesce, Isabelle Pin, Chantal Raherison, Antonio Pereira-Vega, Francisco Gómez Real, Kai Triebner

**Affiliations:** 1 Faculty of Medicine, University of Iceland, Reykjavík, Iceland; 2 Department of Sleep, Landspitali University Hospital, Reykjavík, Iceland; 3 Department of Clinical Science, University of Bergen, Bergen, Norway; 4 Core Facility for Metabolomics, University of Bergen, Bergen, Norway; 5 ISGlobal, Barcelona, Spain; 6 Universitat Pompeu Fabra (UPF), Barcelona, Spain; 7 CIBER Epidomiología y Salud Pública (CIBERESP), Madrid, Spain; 8 University Hospital of Montpellier, IDESP, INSERM-Univ Montpellier, Montpellier, France; 9 Department of Surgical and Perioperative Sciences, Surgery Umeå University, Umeå, Sweden; 10 Institute and Outpatient Clinic for Occupational, Social and Environmental Medicine, Ludwig Maximilians University Munich, Munich, Germany; 11 Allergy and Lung Health Unit, Melbourne School of Population and Global Health, The University of Melbourne, Melbourne, Australia; 12 Department of Occupational and Environmental Medicine, University of Gothenburg, Gothenburg, Sweden; 13 National Heart and Lung Institute, Imperial College London, London, United Kingdom; 14 Tartu University Hospital, Lung Clinic, Tartu, Estonia; 15 Université Paris-Saclay, UVSQ, Univ. Paris-Sud, Inserm, Équipe d’Épidémiologie Respiratoire intégrative, CESP, 94807, Villejuif, France; 16 Department of Medical Sciences, Respiratory, allergy and sleep research, Uppsala University, Uppsala, Sweden; 17 Pulmonology Service, Albacete University Hospital Complex, Health Service of Castilla—La Mancha, Albacete, Spain; 18 Faculty of Medicine of Albacete, Castilla-La Mancha University, Albacete, Spain; 19 Unit of Epidemiology and Public Health, Department of Health, Basque Government, Bilbao, Spain; 20 Department of Paediatrics, University Hospital Grenoble Alpes, French National Institute of Health and Medical Research, Grenoble, France; 21 Institute for Advanced Biosciences, Grenoble; University Grenoble Alpes, 38043, Grenoble cedex 9, France; 22 U1219, Bordeaux Population Health Research, Bordeaux University, 33076, Bordeaux, France; 23 Service of Pneumology and Allergy, University Hospital Juan Ramón Jiménez, Huelva, Spain; 24 Department of Gynaecology and Obstetrics, Haukeland University Hospital, Bergen, Norway; 25 Research Unit for health surveys, Department of Clinical Science, University of Bergen, Bergen, Norway; Brigham and Women’s Hospital and Harvard Medical School, UNITED STATES

## Abstract

**Background:**

The prevalence of obstructive sleep apnea is higher in women after menopause. This is suggested to be a result of an altered sex hormone balance but has so far not been confirmed in a population-based study.

**Objective:**

To investigate whether serum concentration of estrogens and progesterone are associated with the prevalence of sleep apnea symptoms in middle-aged women of the general population.

**Methods:**

We analyzed data from 774 women (40–67 years) from 15 study centers in seven countries participating in the second follow-up of the European Community Respiratory Health Survey (2010–2012). Multiple logistic regression models were fitted with self-reported symptoms of sleep apnea as outcomes and serum concentrations of various estrogens and progesterone as predictors. All analyses were adjusted for relevant covariates including age, BMI, education, study center, smoking habits, and reproductive age.

**Results:**

Among all included women, a doubling of serum concentrations of estrone and progesterone was associated with 19% respectively 9% decreased odds of snoring. Among snorers, a doubling of the concentrations of 17β-estradiol, estrone and estrone 3-sulfate was associated with 18%, 23% and 17% decreased odds of breathing irregularly, and a doubling of the progesterone concentration was further associated with 12% decreased odds of waking up suddenly with a chocking sensation. Other evaluated associations were not statistically significant.

**Conclusions:**

Middle-aged women with low serum estrogen and progesterone levels are more likely to snore and report symptoms of obstructive sleep apnea.

## Introduction

Obstructive sleep apnea (OSA) is characterized by repeated cessations of breathing, caused by upper airway obstruction [1,2]. Apart from substantially lowering quality of life through poor sleep, OSA may increase the production of free radicals with corresponding oxidative stress and have a widespread negative impact on the body, particularly the cardiovascular system [3,4]. OSA is more common in men than in women and epidemiological studies suggest the ratio to be around 2:1 male to female [5–7]. However, the prevalence and severity of OSA increases among women after menopause [8,9]. The prevalence of sleep apnea in postmenopausal women not using hormone replacement therapy is significantly lower than in men after adjusting for age and BMI, namely 2.7% versus 3.9% using the Sleep Disorders Clinic criteria and 5.5% versus 7.2%, using the sleep laboratory criteria [10]. The decreasing sex difference in OSA prevalence with increasing age [11] suggests that the hormonal shift attributed to menopause might play a role in the pathology of OSA. This hypothesis is supported by the finding that women with polycystic ovary syndrome, a disease characterized by comparatively low levels of female sex hormones, are at greater risk for developing sleep apnea [12]. Therefore the main female sex hormone 17β-estradiol, likely plays a role in developing OSA, possibly through its antioxidant properties [13,14]. Additionally, 17β-estradiol receptors are associated with the development of the upper respiratory tract musculature [15,16] and progesterone acts as respiratory stimulant. Experiments in rodents indicate progesterone to exert a protective function against OSA through its role in the hypoxia reflex [14].

Although all these observations are suggestive for a potential impact of female sex hormones on the risk of OSA, to date there is no population-based or large clinical study available; only a handful of smaller studies have reported lower levels of endogenous female sex hormones [17–19] in women with OSA, and there is limited evidence regarding a possible beneficial effect of exogenous sex hormones (e.g. hormone replacement therapy) [9].

With the aging of the general population the time women expect to live after menopause is becoming an ever-increasing portion of their total lifespan. Considering that OSA may lead to cardiovascular conditions (ischemic heart disease and stroke) [20], which are among the greatest contributors to mortality worldwide [21], gaining a broader understanding of the reasons for the increased propensity of postmenopausal women to develop OSA is important. Therefore, we aimed to investigate the association between serum concentration of female sex hormones and sleep apnea symptoms in a large population-based cohort including well-characterized subjects and high-sensitivity hormone measurements.

## Methods

### Study population

**Fig 1:**
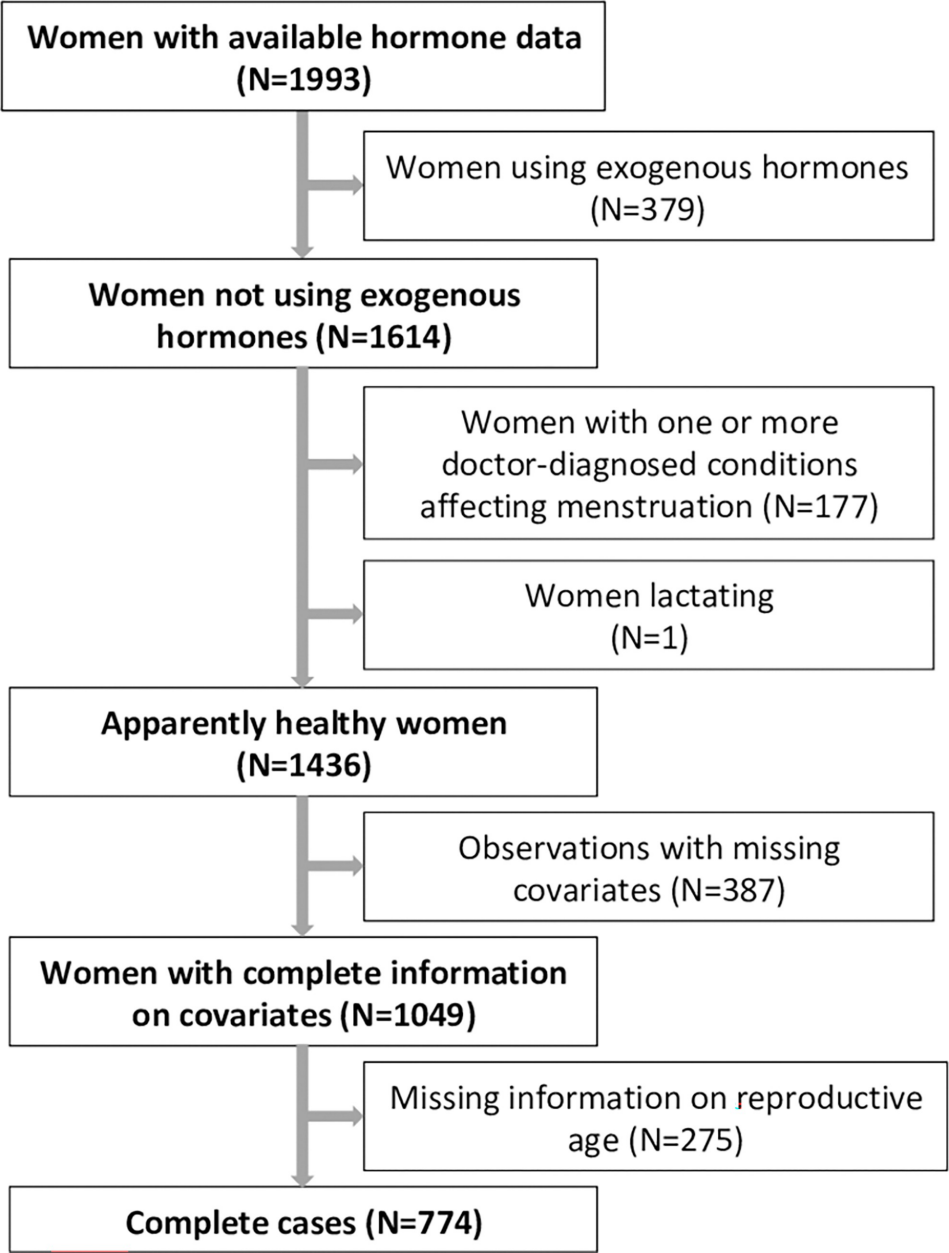
Flowchart of the study population with exclusion criteria.

The analyses were based on 774 complete case observations from the second follow-up (2010–2012) of the European Community Respiratory Health Survey (ECRHS, response rate 49%). Examinations consisted of a structured interviewer-led questionnaire on respiratory health and lifestyle factors; a validated sleep questionnaire [22] and an in-clinic examination where blood samples for hormone analyses were drawn and anthropometric measurements taken. In addition, the female study participants were asked to complete a questionnaire on women’s health. In order to form hormonally well-defined groups we excluded women answering affirmatively to any of the following questions, indicating gynecological conditions: 1)“Have you ever had excessive growth of body hair?”; 2)“Has a doctor or health professional ever told you have polycystic ovaries or polycystic ovary syndrome?”; and 3)“Has a doctor or health professional ever told you have endometriosis”. We further excluded all women taking hormone replacement therapy, hormonal contraception or other exogenous hormone treatment and/or modulators of the genital system for menopausal symptoms, fertility treatment, gynecological disorders or undisclosed reasons. Additional exclusion criteria were current pregnancy, lactation and persistent irregular menstruation, independent of the menopausal transition. We carried out complete case analyses, therefore women with missing data on hormone levels or covariates were also excluded (Fig 1). Data were missing at random. A comparison between the excluded and included study population is presented in S1 Table. The distribution of snorers over traditionally used menopausal categories can be found in S2 Table.

Ethical approval was obtained from the appropriate ethical committee of each study center and all participants provided informed written consent before being enrolled in the study. The specific committees were for Aarhus: Scientific ethical committee for Region Midtlylland; Albacete: Comité Ético de Investigación Clínica del Hospital Universitario de Albacete; Galdakao: Comité Ético de Investigación Clínica del Hospital de Galdakao-Usansolo; Huelva: Comisión de Ética de Investigación Sanitarias del Hospital Juan Ramón Jiménez de Huelva; Bergen: Regional Committee for Medical and Health Research Ethics in Western Norway (2010/759); Bordeaux, Grenoble, Montpellier, Paris: Comite De Protection Des Personnes (2011-A00013-38); Erfurt, Hamburg: Ethikkommission der Bayerischen Landesärztekammer (Reg Nr. 10015); Uppsala, Umeå, Göteborg: Ethics Committee of Uppsala University (2010/068); Reykjavik: National Bioethics Committee of Iceland (VSN 11–121); Tartu: Research Ethics Committee of the University of Tartu (209/T-17);

### Hormone measurements

The serum samples of the participants were analyzed at the Core Facility for Metabolomics at the University of Bergen (Norway) for concentrations of the steroid hormones 17β-estradiol, estrone, estrone 3-sulfate and progesterone, using a high-sensitivity liquid chromatography-tandem mass spectrometry method [23]. The measurement range for estrogens is well in agreement with the reference range in females, only slightly constrained by the analytical sensitivity in the very low range. If a measurement exceeded the upper limit of quantification (ULQ) it was included as 1.5*ULQ. If a measurement was lower than the lower limit of quantification (LLQ) it was included as LLQ/2 [24]. Progesterone could not be evaluated well in the lower range, which was considered in the subsequent statistical analyses.

### Outcome assessment

In the sleep questionnaire women were asked “Have you been told that you snore”, which we used as main outcome (1. Snoring). An affirmative answer lead to three subsequent questions: “In the last 12 months have you been told that you stop breathing or have irregular breathing while you are sleeping?” (2. Irregular breathing); “Have you woken up all of a sudden with a choking sensation or not being able to breathe in the last 12 months?” (3. Gasping); “Have you been told that you snore loudly or that your snoring disturbs other people in the last 12 months?” (4. Disturbing snore). The response options to these questions were: “never”, “seldom”, “sometimes”, “frequently” or “every time”. If women answered “no” to the question “Have you been told that you snore?” they were allotted the answer “never” to all three subsequent questions for the analysis. In order to transform the outcome into a dichotomous variable we merged all affirmative answers (“seldom”, “sometimes”, “frequently” or “every time”).

### Statistical analysis

We fitted logistic regression models with female sex hormones (log_2_-transformed) as predictors (one at a time) and snoring, irregular breathing, gasping and sleep disturbance as outcomes (one at a time). We adjusted the analysis for a predetermined set of covariates: age (continuous), BMI (continuous), smoking habits (lifelong non-smoker, ex-smoker and smoker), age at completed full time education (<17 years, 17–20 years, >20 years) as a socioeconomic proxy, study center and a novel reproductive score (RAS) based on fuzzy logic, which describes the progress of reproductive aging in a continuous manner, taking into account menstrual frequency and age [25]. The distribution of the RAS over the included study population (density plot) and its univariate association with age can be found in S4 Fig.

To evaluate linearity, we carried out sensitivity analyses for quartiles of 17β-estradiol, estrone and estrone 3-sulfate. As the study population contained many postmenopausal women, various progesterone measurements were below LLQ, therefore analyses concerning this hormone were carried out using it as dichotomous variable (below vs. above LLQ). We further evaluated models (based on the main model) that additionally were mutually adjusted for all included hormones (17β-estradiol, estrone, estrone 3-sulfate, progesterone) as well as the use of multi-level models (subjects nested within study centers).

We conducted sensitivity analyses on a) the subset of women who reported to be married and/or cohabiting, and b) adjusting for the frequency of consumption of beer, wine and spirits (Options: “rarely/never”, “1–3 times per month”, “once per week”, “2–4 times per week”, “5–6 times per week”, “once a day”, “2–3 times per day”, “4 or more times per day”). To evaluate the dynamics of the respective sex hormones we additionally conducted sensitivity analyses on hormone ratios. Analyses were performed using R (Version 3.1.0, The R Foundation for Statistical Computing).

## Results

### Study population

**Fig 2:**
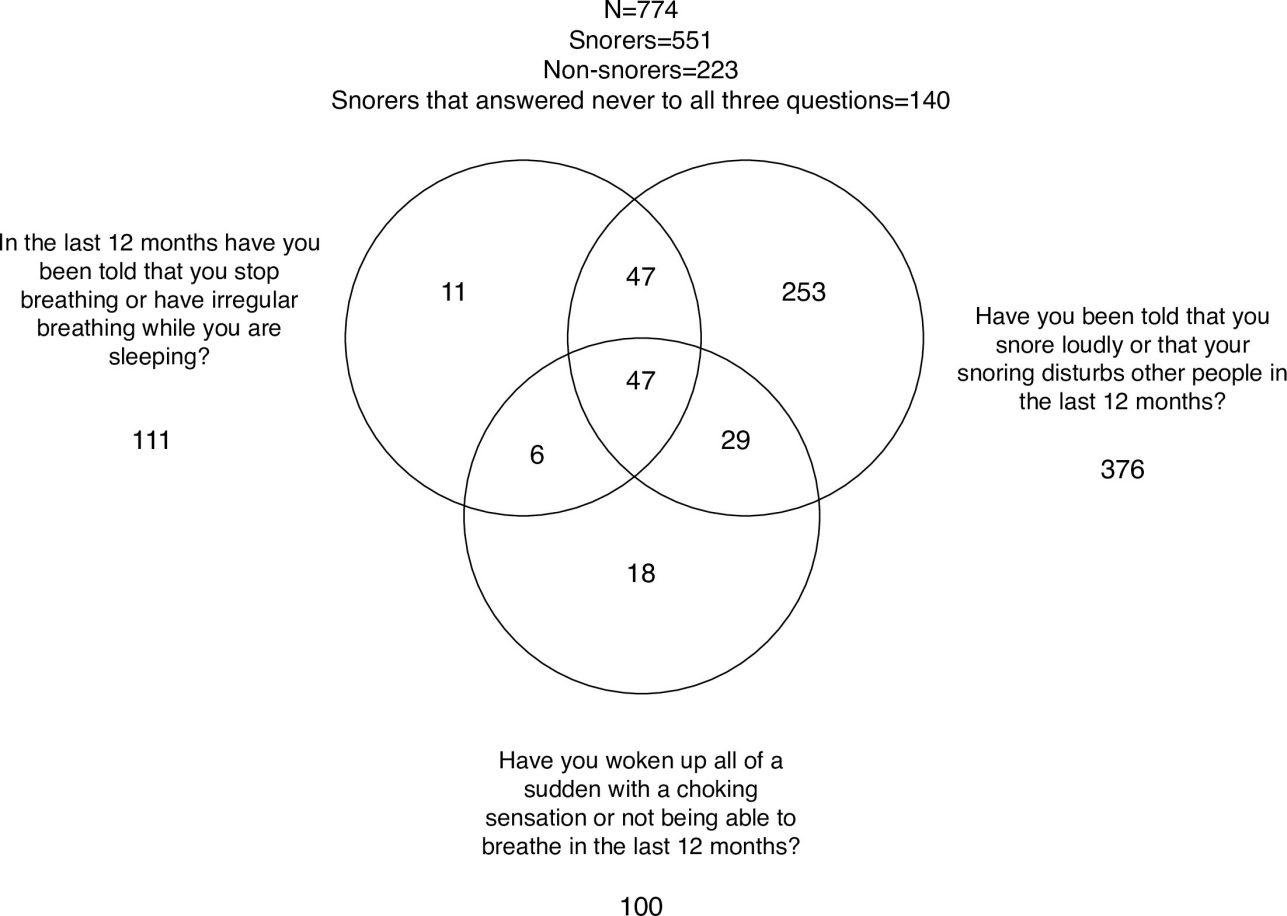
Venn diagram outlining the inter-presence of sleep apnea symptoms.

Anthropometric characteristics of the study population are presented in Table 1. Out of the 774 women, 551 women reported they had been told they snored, of whom 411 responded positively to at least one of the three OSA sub questions (Fig 2). Characteristics of the study population and by reported snoring are presented in Table 2.

**Table 1:** Linear range and quantification limits of the hormone measurements.

Hormone	Linear range^1^, pmol/L	Observations below LLQ^1^	Observations above ULQ^1^
17β-Estradiol	3.64–3 000	57	0
Estrone	2.08–3 000	0	5
Estrone 3-sulfate	242.42–200 000	44	41
Progesterone	212.12–100 000	542	0

^1^Lower limit of quantification (LLQ)–Upper limit of quantification (ULQ).

**Table 2:** Characteristics of the study population and by reported snoring.

	All (N = 774)	Snorers (N = 551)	Non-snorers (N = 223)
**Age (years)**	54.2 (6.97)	54.6 (6.83)	53.1 (7.20)
**BMI (kg/m** ^**2**^ **)**	26.8 (5.37)	27.6 (5.61)	24.9 (4.14)
**Smoking behavior**			
Non-smoker	321 (41.5%)	223 (40.5%)	98 (43.9%)
Ex-smoker	323 (41.7%)	228 (41.4%)	95 (42.6%)
Current smoker	130 (16.8%)	100 (18.1%)	30 (13.5%)
**Age at completed education**			
<17 years	171 (22.1%)	140 (25.4%)	31 (13.9%)
17–20 years	245 (31.7%)	174 (31.6%)	71 (31.8%)
>20 years	358 (46.3%)	237 (43.0%)	121 (54.3%)
**Sleep apnea symptoms**			
Irregular breathing	111 (14.3%)	111 (20.1%)	n/a
Gasping	100 (12.9%)	100 (18.1%)	n/a
Disturbing snore	376 (48.6%)	376 (68.2%)	n/a
**Reproductive parameters**			
Reproductive aging score	0.798 (0.319)	0.819 (0.306)	0.745 (0.345)
17β-Estradiol (pmol/L)	18.1 [2.55, 3940]	17.3 [2.55, 3940]	23.9 [2.55, 2380]
Estrone (pmol/L)	84.4 [3.70, 1500]	81.1 [3.70, 1500]	93.3 [20.0, 1500]
Estrone 3-sulfate (pmol/L)	1260 [171, 15000]	1230 [171, 15000]	1320 [171, 15000]
Progesterone (pmol/L)	150 [150, 61800]	150 [150, 61800]	150 [150, 48900]

n/a: Not applicable.

N (SD) for continuous variables, N (%) for categorical variables and average [interquartile range] for measured serum concentrations.

### Main analysis

**Fig 3:**
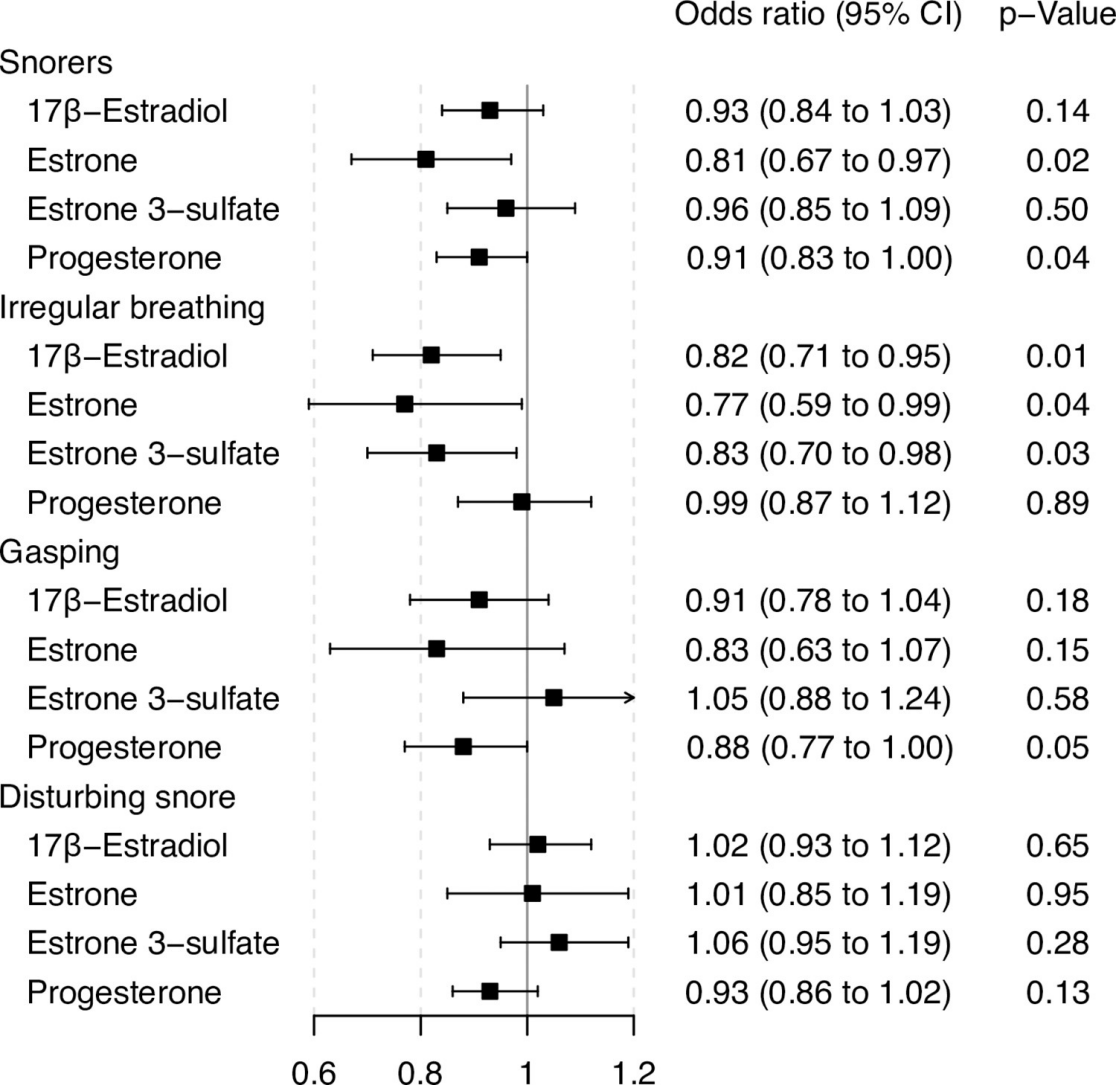
Results of the main analyses, odds ratio and 95% confidence intervals of female sex hormones with obstructive sleep apnea symptoms, adjusted for age, BMI, smoking, age at completed full time education, study center and reproductive aging score.

Among the included study population, a doubling of the concentrations of estrone and progesterone in the bloodstream was associated with 19% respectively 9% decreased odds of snoring. Among women who were told to be snorers, a doubling of the concentrations of 17β-estradiol, estrone and estrone 3-sulfate was associated with 18%, 23% and 17% decreased odds of being told that they stop breathing or breathed irregularly while being asleep (irregular breathing). A doubling of progesterone concentrations was further associated with a 12% decreased chance of waking up suddenly with a choking sensation or not being able to breathe (gasping). Other evaluated associations were not statistically significant (Fig 3).

### Sensitivity analyses

The quartile analyses confirmed the linearity of the associations and while the included sex hormones highly correlate with each other (pairwise correlations: p<0.0001) mutual adjustment resulted in very similar findings (see Table 3) as seen for the main analyses. This was also the case when we applied multilevel model with subjects nested into centers. The sensitivity analyses on a) the subset of women who reported to be married and/or cohabiting, and b) additional adjustment for the frequency of consumption of beer, wine and spirits yielded very similar results as the main model (S1 and S2 Figs). The results of the evaluation of the dynamics using sex hormone ratios showed significant associations between higher ratios of 17β-estradiol to estrone and 17β-estradiol to progesterone with lower odds of irregular breathing. (S3 Fig). A higher ratio of Estrone 3-sulfate to Estrone was associated with lower odds of gasping and a higher ratio of Estrone 3-sulfate to progesterone was associated with higher odds of gasping and disturbing snore.

**Table 3:** Odds ratio and 95% Confidence interval for a model with adjustment for all measured female sex hormones, age, BMI, smoking, age at completed full time education, study center and reproductive aging score.

	OR (95%CI)	p-Value
**Snorers**		
17β-Estradiol	1.07 (0.89 to 1.28)	0.464
Estrone	0.60 (0.41 to 0.88)	0.009
Estrone 3-sulfate	1.23 (0.99 to 1.52)	0.056
Progesterone	0.93 (0.84 to 1.02)	0.121
**Irregular breathing**		
17β-Estradiol	0.78 (0.61 to 1.00)	0.052
Estrone	1.17 (0.70 to 1.93)	0.548
Estrone 3-sulfate	0.92 (0.71 to 1.20)	0.544
Progesterone	1.08 (0.94 to 1.24)	0.303
**Gasping**		
17β-Estradiol	0.95 (0.73 to 1.22)	0.662
Estrone	0.61 (0.36 to 1.03)	0.062
Estrone 3-sulfate	1.47 (1.11 to 1.96)	0.007
Progesterone	0.90 (0.79 to 1.02)	0.108
**Disturbing snore**		
17β-Estradiol	1.06 (0.90 to 1.24)	0.508
Estrone	0.81 (0.58 to 1.13)	0.214
Estrone 3-sulfate	1.17 (0.97 to 1.41)	0.100
Progesterone	0.93 (0.85 to 1.02)	0.106

## Discussion

To our knowledge, this is the first large-scale epidemiological study evaluating the potential influence of female sex hormones on OSA symptoms. We found that women with lower serum concentrations of female sex hormones are more likely to suffer from the following symptoms of OSA: snoring, irregular breathing and gasping. We could not observe statistically significant associations for disturbing snore, which may be due to the individual reception of “disturbing” and/or reporting behavior [26]. The dynamic between sex hormones may be important for the association with OSA symptoms. The sensitivity analyses on hormone ratios, as well as the analysis with mutual adjustment suggest some interplay between estrone and its sulfated form estrone 3-sulfate as well as between 17β-estradiol and estrone, which can be easily converted into each other. However, in the current study we cannot draw profound conclusions on this dynamic due to its complexity.

With increasing (reproductive) age, sex hormone levels are expected to be correspondingly lower, therefore the observed results need to be interpreted with baseline sex hormone levels in mind.

While there are no other population-based epidemiological studies on our evaluated associations, our observed results are in line with existing small clinical and pre-clinical studies, indicating a protective effect of female sex hormones [17–19,27,28] Estrogens have been found to play a role in a diverse set of physiological processes and metabolism, not only limited to female reproductive health and behavior. Both high and low levels of estrogens have been linked to various diseases including those of autoimmune, metabolic and neural nature [29]. The role of estradiol is well established in the development of muscles in the upper respiratory tract [15] as well as a determinant of fat distribution [16], which are both plausible mechanisms where low levels of estrogens could predispose women to report OSA symptoms. That low levels of estrogens are associated with decreased odds of symptoms of OSA is also in line with recent findings indicating that estradiol may mitigate the effects of sleep apnea [13,14] Estradiol has been shown to have an antioxidant effect [28]. It increases the levels of thioredoxin, a protein with antioxidant properties, and moderates the expression of HIF-1α (Hypoxia Induced Factor). Through these mechanisms estrogens might exert their antioxidant functions [28]. As OSA is a disease characterized by intermittent periods of hypoxia resulting in increased oxidant stress and the antioxidant activity of estrogens might mitigate its negative effects. Pre-clinical studies further show that estrogens as well as phytoestrogens attenuate the harmful effects of chronic intermittent hypoxia (OSA in animals) on the genioglossal muscles in ovariectomized rodents, supposedly by attenuating lung inflammation, bronchoconstriction and increasing antioxidant defense [28]. Clinical studies on postmenopausal women, agree that treatment with female sex hormones could have beneficial effects on obstructive apnea [9,27–31] Progesterone on the other hand is a well-known respiratory stimulant with capacity to reduce the frequency of apneas, even though the underlying mechanisms remain unclear. Recent studies in mice have shown that the respiratory effects of progesterone are mediated by at least two classes of progesterone receptors, which may have sex-specific effects on the frequency of apneas [14].

A major strength of our study was the use of a well-characterized general population sample and the use of the novel RAS allowed for a thorough continuous adjustment for reproductive aging [25]. Using the continuous RAS in epidemiological studies is advantageous over using heterogeneous categories of menopause such as “nonmenopausal”, “peri-menopausal” and “postmenopausal”, as it reduces classification errors, increases statistical power of the regression analyses and is easily obtainable. The detailed women’s questionnaire also enabled the exclusion of women with likely abnormal hormonal profiles due to endocrine gynecological conditions such as polycystic ovary syndrome and endometriosis as well as the use of hormonal medication. A further strength was the use of objective hormone measurements obtained with a state-of-the-art high sensitivity LC-MS/MS method. Another strength was the fact that this study was conducted in seven countries across different cultures, lifestyles and environments across Europe. A limitation to our study was its cross-sectional nature, restricting meaningful statements on causality. The current study evaluated four self-reported symptoms of nocturnal breathing and awakenings. Therefore it carries its limitations, but literature suggests the frequency of these symptoms among female OSA patients is sufficient to validate its findings. We further did not have data to evaluate the potential influence of hormonal variations throughout the menstrual cycle, circadian rhythmicity, nutritional status or stress exposure.

To better the understanding of the dynamics of the condition and the underlying biology, longitudinal studies including clinical evaluation of the condition are highly warranted. We further suggest repeated hormone profiling and increased focus on more female-specific symptoms, such as fatigue, morning headaches and insomnia [32].

## Conclusions

We observed that in middle-aged women lower levels of female sex hormone were associated with higher risk of suffering from OSA symptoms. It is crucial to develop strategies to decrease the high prevalence and associated morbidity of OSA and adjusting female sex hormones levels might be the key to accomplish this, but further longitudinal studies with repeated measures of sex hormones and objective characterization of OSA are required to confirm our findings in other settings.

## Supporting information

S1 Table Comparing included and excluded women. Mean (SD) for continuous variables, N (%) for categorical variables and mean [interquartile range] for measured hormone concentrations.
(DOCX)

S2 Table Distribution of snorers over traditionally used menopausal categories. (DOCX)

S1 Fig Results of the sensitivity analyses among married/cohabiting women (N = 580), odds ratio and 95% confidence intervals of female sex hormones with obstructive sleep apnea symptoms, adjusted for age, BMI, smoking, age at completed full time education, study center and reproductive aging score. (DOCX)

S2 Fig Results of the sensitivity analyses considering alcohol intake (N = 433), odds ratio and 95% confidence intervals of female sex hormones with obstructive sleep apnea symptoms, adjusted for age, BMI, smoking, age at completed full time education, study center, reproductive aging score and frequency of consumption of beer, wine and spirits. (DOCX)

S3 Fig Results of the analyses using hormone ratios as predictors, odds ratio and 95% confidence intervals of female sex hormones with obstructive sleep apnea symptoms, adjusted for age, BMI, smoking, age at completed full time education, study center and reproductive aging score. (DOCX)

S4 Fig Distribution of the Reproductive Aging Score (RAS) over the included study population (density plot) and its univariate association with age (linear General Additive Model). (DOCX)

## Abbreviations

CI: Confidence interval
LC-MS/MS: Liquid chromatography-tandem mass spectrometry
LLQ: Lower Limit of quantification
ULQ: Upper Limit of quantification
OSA: Obstructive Sleep Apnea
ECRHS: European Community Respiratory Health Survey
