# Supplementary material for: Female sex hormones and symptoms of obstructive sleep apnea in European women of a population-based cohort
Source: PLoS One. 2022 Jun 22;17(6):e0269569. doi: 10.1371/journal.pone.0269569 (PMC9216532; doi:10.1371/journal.pone.0269569)
Supplement: S1 Table — Mean (SD) for continuous variables, N (%) for categorical variables and mean [interquartile range] for measured hormone concentrations. (DOCX) [file pone.0269569.s001.docx]

S1 table. Comparing included and excluded women. Mean (SD) for continuous variables, N (%) for categorical variables and mean [interquartile range] for measured hormone concentrations.

|  | **Excluded** | **Included** | **p-Value^1^** |
| --- | --- | --- | --- |
| **N** | 1045 | 774 |  |
| **Age (years)** | 54.5 (6.9) | 54.2 (7.0) | 0.281 |
| **BMI (kg/m^2^)** | 26.7 (5.0) | 26.8 (5.4) | 0.646 |
| **Smoking habits** |  |  | 0.962 |
| Lifelong nonsmoker | 433 (42.0) | 321 (41.5) |  |
| Ex-smoker | 428 (41.6) | 323 (41.7) |  |
| Smoker | 169 (16.4) | 130 (16.8) |  |
| **Age at completed education (years)** | |  | 0.015 |
| <17 years | 175 (16.7) | 171 (22.1) |  |
| 17-20 years | 345 (33.0) | 245 (31.7) |  |
| >20 years | 525 (50.2) | 358 (46.3) |  |
| **Sleep apnea symptoms** |  |  |  |
| Snorers | 744 (72.2) | 551 (71.2) | 0.664 |
| Irregular breathing | 158 (15.4) | 111 (14.3) | 0.566 |
| Gasping | 137 (13.3) | 100 (12.9) | 0.861 |
| Disturbing snore | 535 (52.0) | 376 (48.6) | 0.165 |
| **Reproductive parameters** |  |  |  |
| Reproductive aging score | 1.0 [0.8, 1.0] | 1.0 [0.7, 1.0] | 0.865 |
| 17β-Estradiol (pmol/L) | 17.9 [8.4, 134.8] | 18.1 [8.2, 157.3] | 0.962 |
| Estrone (pmol/L) | 83.3 [55.5, 156.8] | 84.3 [54.1, 162.0] | 0.806 |
| Estrone 3-sulfate (pmol/L) | 1191.4 [611.2, 3179.1] | 1263.0 [609.1, 3282.5] | 0.852 |
| Progesterone (pmol/L) | 149.9 [149.9, 229.1] | 149.9 [149.9, 297.3] | 0.051 |

^1^Chi squared test for categorical variables and Mann-Whitney U test for continuous variables
