# Supplementary material for: Female sex hormones and symptoms of obstructive sleep apnea in European women of a population-based cohort
Source: PLoS One. 2022 Jun 22;17(6):e0269569. doi: 10.1371/journal.pone.0269569 (PMC9216532; doi:10.1371/journal.pone.0269569)
Supplement: S2 Table — (DOCX) [file pone.0269569.s002.docx]

S2 table. Distribution of snorers over traditionally used menopausal categories

|  | **Nonmenopausal** | **Peri-menopausal** | **Menopausal** |
| --- | --- | --- | --- |
| **Non-snorers** | 60 (39%) | 27 (31%) | 125 (25%) |
| **Snorers** | 95 (61%) | 60 (69%) | 375 (75%) |
