# Supplementary material for: Female sex hormones and symptoms of obstructive sleep apnea in European women of a population-based cohort
Source: PLoS One. 2022 Jun 22;17(6):e0269569. doi: 10.1371/journal.pone.0269569 (PMC9216532; doi:10.1371/journal.pone.0269569)
Supplement: S2 Fig — (DOCX) [file pone.0269569.s004.docx]

**S2 fig. Results of the sensitivity analyses** considering **alcohol intake** (N=433), odds ratio and 95% confidence intervals of female sex hormones with obstructive sleep apnea symptoms, adjusted for age, BMI, smoking, age at completed full time education, study center, reproductive aging score and frequency of consumption of beer, wine and spirits.
