# Supplementary material for: Female sex hormones and symptoms of obstructive sleep apnea in European women of a population-based cohort
Source: PLoS One. 2022 Jun 22;17(6):e0269569. doi: 10.1371/journal.pone.0269569 (PMC9216532; doi:10.1371/journal.pone.0269569)
Supplement: S3 Fig — (DOCX) [file pone.0269569.s005.docx]

S3 fig. Results of the analyses using hormone ratios as predictors, odds ratio and 95% confidence intervals of female sex hormones with obstructive sleep apnea symptoms, adjusted for age, BMI, smoking, age at completed full time education, study center and reproductive aging score.
