# Supplementary material for: Female sex hormones and symptoms of obstructive sleep apnea in European women of a population-based cohort
Source: PLoS One. 2022 Jun 22;17(6):e0269569. doi: 10.1371/journal.pone.0269569 (PMC9216532; doi:10.1371/journal.pone.0269569)
Supplement: S4 Fig — (DOCX) [file pone.0269569.s006.docx]

S4 fig. Distribution of the Reproductive Aging Score (RAS) over the included study population (density plot) and its univariate association with age (linear General Additive Model)
